# Supplementary material for: Internet of things–Enabled technologies as an intervention for childhood obesity: A systematic review
Source: PLOS Digit Health. 2022 Apr 7;1(4):e0000024. doi: 10.1371/journal.pdig.0000024 (PMC9931243; doi:10.1371/journal.pdig.0000024)
Supplement: S1 Fig — (DOCX) [file pdig.0000024.s002.docx]

APPENDIX 1


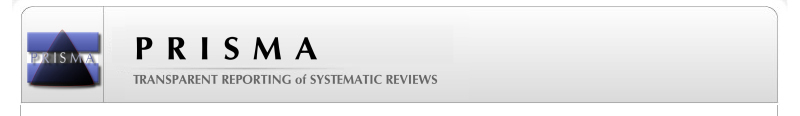
PRISMA 2009 Flow Diagram

Full-text articles excluded, with reasons
(no IoT intervention = 76

Full paper identified = 2

Full text not found = 1

Not a study/paper =1

Not specific for youth = 7

Not for weight management = 3

Not in English = 1

Review = 1)

Additional records identified through other sources:
From references
(n = 2 )

Records identified through database searching
(n = 2541 )

Studies included in quantitative synthesis (meta-analysis)
(n = 23 )

Studies included in qualitative synthesis
(n = 23 )

Full-text articles assessed for eligibility
(n = 115 )

Records excluded
(n = 2359 )

Records screened
(n = 2474 )

Records after duplicates removed
(n = 2474 )

## Identification

## Eligibility

## Included

## Screening
