# Supplementary material for: Internet of things–Enabled technologies as an intervention for childhood obesity: A systematic review
Source: PLOS Digit Health. 2022 Apr 7;1(4):e0000024. doi: 10.1371/journal.pdig.0000024 (PMC9931243; doi:10.1371/journal.pdig.0000024)
Supplement: S1 Table — (DOCX) [file pdig.0000024.s003.docx]

S1 Table. Included Studies

| Study ID | Studies included | Study Title | Ref |
| --- | --- | --- | --- |
| 1 | Alahmadi 2013 | Electronic monitor for monitoring TV viewing time description and significance | [13] |
| 2 | Alloghani 2016 | A Mobile Health Monitoring Application for Obesity Management and Control Using the Intemet-of-Things | [14] |
| 3 | Bi  2017 | FamilyLog: A mobile system for monitoring family mealtime activities | [15] |
| 4 | Caon  2018 | Teenagers’ Usage of a Mobile-Wearable-Cloud Platform to Promote Healthy Lifestyles: the PEGASO Experience | [16] |
| 5 | De Cock 2016 | Feasibility and impact study of a reward-based mobile application to improve adolescents' snacking habits | [17] |
| 6 | Delopoulos 2019 | Big Data Against Childhood Obesity, the BigO Project | [18] |
| 7 | Direito  2015 | Apps for IMproving FITness and increasing physical activity among young people: The AIMFIT pragmatic randomized controlled trial | [19] |
| 8 | Garde  2015 | Assessment of a Mobile Game ("MobileKids Monster Manor") to Promote Physical Activity Among Children | [20] |
| 9 | Lindberg 2016 | Enhancing Physical Education with Exergames and Wearable Technology | [21] |
| 10 | Lopez  2017 | MATCHuP: An mhealth tool for children and young people health promotion | [22] |
| 11 | Lu  2013 | Reducing adolescent obesity with a mobile fitness application: Study results of youth age 15 to 17 | [23] |
| 12 | Maramis 2014 | Preventing Obesity and Eating Disorders through Behavioural Modifications: the SPLENDID Vision | [24] |
| 13 | Mendoza 2017 | A Fitbit and Facebook mHealth intervention for promoting physical activity among adolescent and young adult childhood cancer survivors: A pilot study | [25] |
| 14 | O'Malley 2014 | A smartphone intervention for adolescent obesity: study protocol for a randomised controlled non-inferiority trial | [26] |
| 15 | Phan  2018 | Feasibility of using a commercial fitness tracker as an adjunct to family-based weight management treatment: Pilot randomized trial | [27] |
| 16 | Ridgers 2017 | A cluster-randomised controlled trial to promote physical activity in adolescents: The Raising Awareness of Physical Activity (RAW-PA) Study | [28] |
| 17 | Ridgers  2018 | Wearable activity tracker use among Australian adolescents: Usability and acceptability study | [29] |
| 18 | Svensson 2015 | A Mobile Phone App for Dietary Intake Assessment in Adolescents: An Evaluation Study | [30] |
| 19 | Taki  2019 | A Mixed Methods Study to Explore the Effects of Program Design Elements and Participant Characteristics on Parents' Engagement With an mHealth Program to Promote Healthy Infant Feeding: The Growing Healthy Program | [31] |
| 20 | Turel  2016 | Health Outcomes of Information System Use Lifestyles among Adolescents: Videogame Addiction, Sleep Curtailment and Cardio-Metabolic Deficiencies | [32] |
| 21 | Yang  2017 | Interventions for Preventing Childhood Obesity with Smartphones and Wearable Device: A Protocol for a Non-Randomized Controlled Trial | [33] |
| 22 | Tripicchio 2017 | Technology Components as Adjuncts to Family-Based Pediatric Obesity Treatment in Low-Income Minority Youth | [34] |
| 23 | Vazquez-Briseno 2012 | A proposal for using the internet of things concept to increase children's health awareness | [35] |
