# Supplementary material for: Internet of things–Enabled technologies as an intervention for childhood obesity: A systematic review
Source: PLOS Digit Health. 2022 Apr 7;1(4):e0000024. doi: 10.1371/journal.pdig.0000024 (PMC9931243; doi:10.1371/journal.pdig.0000024)
Supplement: S4 Table — (DOCX) [file pdig.0000024.s006.docx]

|  | Institutional review board | Participant consent | Permission at target school | Declaration  of Helsinki | N/A |
| --- | --- | --- | --- | --- | --- |
| Alahmadi 2013 |  |  |  |  | * |
| Alloghani 2016 |  |  |  |  | * |
| Bi 2017 | * |  |  |  |  |
| Caon 2018 | * |  |  |  |  |
| De Cock 2016 | * | * |  |  |  |
| Delopoulos 2019 | * |  |  |  |  |
| Direito 2015 | * | * |  |  |  |
| Garde 2015 | * | * |  |  |  |
| Lindberg 2016 |  |  | * |  |  |
| Lopez 2017 |  |  |  |  | * |
| Lu 2013 |  |  |  |  | * |
| Maramis 2014 |  |  |  |  | * |
| Mendoza 2017 | * | * |  |  |  |
| O'Malley 2014 | * |  |  |  |  |
| Phan 2018 | * | * |  |  |  |
| Ridgers 2018 | * |  |  |  |  |
| Ridgers, 2017 | * |  |  |  |  |
| Svensson 2015 | * | * |  | * |  |
| Taki 2019 | * | * |  |  |  |
| Turel 2016 | * | * |  |  |  |
| Yang 2017 | * | * |  |  |  |
| Tripicchio 2017 | * | * |  |  |  |
| Vazquez-Briseno 2012 |  |  |  |  | * |
