# Supplementary material for: Internet of things–Enabled technologies as an intervention for childhood obesity: A systematic review
Source: PLOS Digit Health. 2022 Apr 7;1(4):e0000024. doi: 10.1371/journal.pdig.0000024 (PMC9931243; doi:10.1371/journal.pdig.0000024)
Supplement: Attachment — Submitted filename: Response to Reviewers (IoT).docx [file pdig.0000024.s008.docx]

| PLOS Digital Health |  |  |
| --- | --- | --- |

17 February 2022

Dear Shelagh,

Thank you for your consideration of our manuscript “Internet of things-enabled technologies as an intervention for childhood obesity: A Systematic Review” for publication in PLOS Digital Health. We have made amendments to the manuscript reflecting the valuable review comments forwarded and feel the paper has been well enhanced to be re-presented for publication. Please let us know if there is anything we can further do to clarify or extend our paper.

With kind regards,

Edward Meinert

Enclosures:

1. Chart mapping peer review feedback

| **ID** | **Reviewer** | **Reviewer Feedback** | **Author response** | **Action taken** |
| --- | --- | --- | --- | --- |
| 1 | Editor | The manuscript has several strengths including standardized rubrics, inclusion of the use of empirically-supported behavior change methods, and substantial supporting documentation for processes and outcome of the study. | We thank the editor for their time and consideration in the review of our manuscript. The raised issues have been addressed in view of the peer review feedback provided. | N/A |
| 2 | Editor | In the abstract, the stated purpose of the review includes evaluation of feasibility and effectiveness but does not include evaluation of ‘system designs’ (data processes, networks) as stated later in the paper. | We appreciate the editor pointing out this oversight and have added systems designs to the abstract. | Abstract edited |
| 3 | Editor | The authors include stakeholders involved in studies, but who were actually the users of the systems? Were stakeholders involved in any aspect of design but not the final intended users? | We have clarified the text in section D to demonstrate that the stakeholders considered used the app for a variety of purposes (detailed in Appendix 4). | Text added |
| 4 | Editor | Please explain why 4 studies had no participants for an intervention study. | We apologise for any confusions; although one of the outcomes examined was the effectiveness measures used and reported effectiveness of the IoT devices, the eligibility criteria did not limited included studies to intervention evaluations. We chose not to limit the type of study included so that we would capture a wider range of papers examining the types of weight management technologies and products available and their potential benefits and limitations. We have clarified this in the eligibility criteria. | Eligibility criteria clarified |
| 5 | Editor | A suggestion to improve the readability of the tables: Table V does not provide a clear indication of how many studies used a particular outcome. Better explanation in the text may also help with that table. | We thank the editor for the suggestion and have clarified the number of studies reporting particular outcomes in text. | Text added |
| 6 | Editor | If the authors would like to put forward the recommendation for developers to focus on gamification for engagement, please add additional support/rationale from the research literature and what features have been found to be most helpful. | In line with the editor (and reviewers’) comments, we have revised the future recommendations section to better reflect the state of the literature and emphasise that gamification is a potentially useful tool to increase engagement that should be explored in interventions for children and young people, but that it requires further empirical support and investigation of long-term impacts. | Text and citations added |
| 7 | Editor | The implications of the results do not come through in the Discussion. What are the implications of the data transfer methods or analyses used to provide data summaries to users? Please describe the possible negative impacts of gamification if social networking is included. Please recommend how future literature reviews could aggregate and organize heterogeneous studies to provide more generalizable results. | We thank the editor for raising this question, we have added a paragraph in the discussion to address the gap identified. We have also added recommendations to address the heterogeneity we identified in the future recommendations. | Paragraphs added |
| 8 | Reviewer 1 | The authors review novel interventions using internet-connected devices to tackle the important problem of childhood obesity. They review studies focusing on a wide variety of devices, including wristbands and smartphone apps, and present the technologies, data analysis processes and effectiveness in an accessible manner. The precise reporting of what was measured and evaluated for various stakeholders in each study in the use case matrix is appreciated - this will enable interested readers to understand the approaches, strengths and weaknesses at a glance. | We thank the reviewer for their time and consideration in the review of our manuscript. The raised issues have been addressed in view of the peer review feedback provided. | N/A |
| 9 | Reviewer 1 | The authors correctly note that game-based interventions appear to result in better engagement, and there is insufficient evidence to show good engagement for non-game-based technologies in children. However, their recommendation to ‘… concentrate on low-cost but engaging game-based technologies’ fails to mention the fact that potential harms have not been systematically evaluated - devices used to bring these interventions to children can also expose them to other games and applications like social networks, which can have adverse effects on physical and mental health, as well as social relationships. It may well be that these game-based technologies will prove to be beneficial on balance, but their future evaluation should not skip over the consideration of these risks in real world settings. While a detailed discussion of these issues is probably beyond the scope of this work, it is important that readers understand that the potential downsides of these approaches have not been studied in the reviewed papers. | As described in ID 6, we have revised the future recommendations section to better reflect the state of the literature and highlight the importance of examining potential unintended consequences of gamification in future research. | Text and citations added |
| 10 | Reviewer 2 | This original research article “Internet of things-enabled technologies as an intervention for childhood obesity: A Systematic Review” is a well written systematic review aiming to assess the current evidence regarding utility of network-connected health Internet of Things (IoT) technologies (smartphones, wearables, sensors, apps, etc) in children and adolescents as a tool for managing and preventing childhood obesity. They appropriately comment on the mental and physical health problems that come along with childhood obesity and the downstream effects it has. They comment on clinic-based interventions having favorable impacts, but note that in person counseling may not be feasible for children in rural areas or with financial difficulties. They propose utilizing IoT devices as a tool for childhood obesity and a solution for location and financial barriers to in person diet/lifestyle counseling. Their analysis involves 23 studies from 2010-2019. The results highlight the most used devices (smartphone/mobile apps) and the most commonly tracked data (physical activity data, accelerometer data). Results indicate that adherence to IoT based approaches was low but improved with game-based interfaces. They conclude that future IoT enabled weight management technologies aimed at children should focus on low-cost, game-based intervention (“gamification”). | We thank the reviewer for their time and consideration in the review of our manuscript. The raised issues have been addressed in view of the peer review feedback provided. | N/A |
| 11 | Reviewer 2 | The premise of this paper is interesting and addresses a very important topic in the health of children and focus on early intervention for childhood obesity and preventive medicine.  The methods used are appropriate, though search criteria may have been restrictive and excluded potentially relevant studies ([link](https://eur03.safelinks.protection.outlook.com/?url=https%3A%2F%2Fpubmed.ncbi.nlm.nih.gov%2F25617572%2F&amp;data=04%7C01%7Cmadison.milne-ives%40plymouth.ac.uk%7C8eafe0d82f724178f6c608d9da81d200%7C5437e7eb83fb4d1abfd3bb247e061bf1%7C1%7C0%7C637781073383278613%7CUnknown%7CTWFpbGZsb3d8eyJWIjoiMC4wLjAwMDAiLCJQIjoiV2luMzIiLCJBTiI6Ik1haWwiLCJXVCI6Mn0%3D%7C3000&amp;sdata=OCZZt4dBM5aOEfdpmM%2FgzKPK6%2FKsmqq0SeAEtk%2FeSdI%3D&amp;reserved=0)), particularly regarding exer-gaming technologies such as Wii-Fit. With the emphasis placed on gamification of future IoT enabled weight management technologies, the omission of interactive gaming consoles, such as Wii-Fit should be strongly considered. | The authors appreciate the reviewer’s perspective and found the linked article interesting. The study protocol did not have a particular focus on gamification or exergames; however, given the potential of gamification as a tool that was identified through the analysis of included papers, we agree that further investigation of the intersection between IoT, gamification, and childhood weight management would be worthwhile. | N/A |
| 12 | Reviewer 2 | One of the major conclusions from the study is that game-based technologies showed superiority, however, their analysis involves very few studies that evaluated gamification as a technique (Per Table IV). Based on the inclusion criteria noted in the paper, there are studies on devices aimed specifically exer-gaming devices (such as Wii-Fit) in adolescents that would be important to include in this analysis (see pub med link in paragraph above) | As mentioned above, the potential benefits of game-based technologies arose from the analysis of the included studies, it was not a particular area of focus when the search strategy and aims were defined. We agree that this would be an important avenue for more in-depth research, on the basis of the results we have found here, which we agree are limited by the low number of included IoT studies that used gamification techniques. We have revised a sentence in the limitations section to reflect this. | Text changed |
| 13 | Reviewer 2 | Table V is very informative but difficult to for the reader to get though. Is there another way to represent these data?  Potentially consolidate? | As suggested by the editor, we have improved the text summary to better reflect the numbers of studies examining particular outcomes to provide a more concise and readable overview of Table V. We have chosen to leave the detail in the table for readers who would like a more in-depth summary. | Text changed |
| 14 | Reviewer 2 | Citation needed at the end of the first Introduction paragraph | We have added two citations to provide evidence for the sentence in question. | Citations added |
| 15 | Reviewer 2 | Introduction, second paragraph, end of second sentence:  "apps" should be spelled out as applications (apps) and then shorthand may be used after. | We thank the reviewer for pointing out this abbreviation and have adjusted accordingly. | Text changed |
| 16 | Reviewer 2 | Generally high use of acronyms and jargon.  To improve readability, this should be clearly spelled out at first use each time. | We thank the reviewer for identifying this issue; we have reviewed the full text to ensure that all acronyms were defined at first use. | Acronyms defined throughout paper |
| 17 | Reviewer 2 | Table I lists the titles of all 23 articles and is better suited for supplemental tables/Appendix rather than in the main text | We appreciate the suggestion and have moved Table 1 to the Appendices. | Appendix added |
| 18 | Reviewer 2 | Figures 1, 2, 3, 4, 5, and 6:  Sort categories in graph by number (either ascending or descending; choose one and stick to that for remainder of bar graphs). Consider grouping categories with only 1 into one bar and specify "one each for the following:  ____" | We appreciate the reviewer’s feedback about the clarity of the figures and have redone them based on the reviewer’s suggestions. | Figures redone |
| 19 | Reviewer 2 | Figure 7 uses a new graph type to display the same data type. The circles are difficult to read. Consider the same chart/display for the same data type. |  |  |
| 20 | Reviewer 2 | Table VI does not add much to the aims of the manuscript—consider moving to supplemental information if included | We appreciate the suggestion and have moved Table VI to the Appendices. | Appendix added |
| 21 | Reviewer 2 | Paragraph right before "Strengths and limitations" header in discussion comments on the lack of adherence to CONSORT e-Health standards and importance of sticking to it. This is a good, and important, opportunity to spread knowledge. I.e. What is CONSORT e-Health and when should it be used. | We thank the reviewer for this suggestion and have added to the paragraph to explain the importance of the CONSORT standards and provided a citation to direct readers to further information about it. | Sentences and citation added |
| 22 | Reviewer 3 | The study addressed a very important issue, to manage children obesity through IoT but the reviewer opinion is that the manuscript cannot be published as it was submitted. It is not clear why most of the papers were discarded, what inclusion criterium they didn’t meet to be discarded. Tables are confused, long, difficult to read. It is not clear how they got the conclusion that gameable IoT are the most effective but decline of engagement was observed. It would be important to include in the conclusion what the goals of the study were, only change of weight? Change of lifestyle? Change of behavioral aspects? It is important to mention in the conclusion and perspectives if the studies were designed to increase or evaluate adherence and long-term effects. Is there any other way to present the information in more readable tables? | We thank the reviewer for their time and consideration in the review of our manuscript. We have moved some of the tables to the appendices and improved their in-text descriptions. We have also revised the recommendations for use of gamification to emphasise that it is a potentially useful tool, as indicated by our results, but one that requires further empirical evaluation. We have added a sentence to the conclusion regarding the aims of the studies and the outcomes examined. | Sentences added |
